# Epigenetics in blood–brain barrier disruption

**DOI:** 10.1186/s12987-021-00250-7

**Published:** 2021-04-06

**Authors:** Stephanie A. Ihezie, Iny Elizebeth Mathew, Devin W. McBride, Ari Dienel, Spiros L. Blackburn, Peeyush Kumar Thankamani Pandit

**Affiliations:** The Vivian L. Smith Department of Neurosurgery, University of Texas Health Science Center, 6431 Fannin St. MSB 7.147, Houston, TX 77030 USA

**Keywords:** Blood–brain barrier, Endothelial cells, Epigenetics, DNA methylation, Histone modifications

## Abstract

The vessels of the central nervous system (CNS) have unique barrier properties. The endothelial cells (ECs) which comprise the CNS vessels contribute to the barrier via strong tight junctions, specific transporters, and limited endocytosis which combine to protect the brain from toxins and maintains brain homeostasis. Blood–brain barrier (BBB) leakage is a serious secondary injury in various CNS disorders like stroke, brain tumors, and neurodegenerative disorders. Currently, there are no drugs or therapeutics available to treat specifically BBB damage after a brain injury. Growing knowledge in the field of epigenetics can enhance the understanding of gene level of the BBB and has great potential for the development of novel therapeutic strategies or targets to repair a disrupted BBB. In this brief review, we summarize the epigenetic mechanisms or regulators that have a protective or disruptive role for components of BBB, along with the promising approaches to regain the integrity of BBB.

## Introduction

Long-term gene expression programs during CNS development are directed by epigenetic mechanisms such as DNA methylation or hydroxymethylation and histone modifications [1]. Recent studies have identified additional epigenetic mechanisms like microRNAs, long non-coding RNAs, and histone variants [1, 2]. These epigenetic mechanisms are known to be influenced by the environment and experience [3].

The vascular network includes arteries and arterioles which distribute blood to the tissues, a fine network of capillary beds that supply essential nutrients and gases inside the tissue, and venules and veins which collect deoxygenated blood from tissues. Vascular properties differ, depending on the needs of the specific organs they vascularize. To meet the unique requirements of the CNS, specialized capillaries in the brain exhibits unique barrier characteristics which have been termed the blood–brain barrier (BBB). The BBB regulates the exchange of molecules between the blood and brain, thus managing the brain environment for vital functions. The CNS vessels are in contact with two immune cell populations one within the blood and the other in the CNS thought to regulate the BBB properties in response to an injury or infection. The CNS immune cell population includes macrophages and microglial cells [4]. Although it is protective, this selective barrier makes an obstacle for CNS drug delivery and significant research efforts have been made to create methods to open this barrier for drug delivery. Aging and conditions, such as hypertension and cerebrovascular ischemia, can aggravate the BBB, thereby changing the BBB components [5, 6], and can contribute to BBB disruption that predisposes the brain to neurological disease including Alzheimer's disease [7, 8]. Further, BBB disruption is a serious concern in many neurological diseases such as stroke and TBI [9–11]. This review covers the various ways in which epigenetic dysregulations contribute to BBB disruption and the epigenetic programs that are modified due to BBB disruption.

## Epigenetic pathways of gene regulation

Epigenetic modifications are chemical modifications occurring in chromatin, DNA, or transcribed RNA that can influence gene expression or activity without changing the DNA sequence [12, 13]. Conventionally, these modifications affect histone proteins, DNA, and/or chromatin remodeling, but also the non-coding RNAs that can regulate the gene expression post-transcriptionally in response to various environmental cues belong to this group [14, 15]. Epigenetic modifications are heritable in nature, stage, and tissue-specific, and involved in global gene silencing to support the normal developmental processes.

### DNA methylation

DNA, the basic unit of heredity is epigenetically modified by methylation. DNA methylation regulates the chromatin state and the accessibility of DNA to the transcription machinery. DNA methylation is carried out by a group of enzymes called DNA methyltransferases (DNMTs). DNMTs catalyze the covalent transfer of a methyl group from S-adenosyl methionine to the cytosine residue present in the CpG dinucleotides [16, 17]. DNMTs can be de novo methylase or maintenance methylase. De novo methylases are responsible for establishing the early methylation pattern during germ cell and embryo development [18]. De novo methylation is catalyzed by the redundant activities of DNMT3a and DNMT3b. Parallel to their redundant activity, each of these methyltransferases has unique targets. DNMT3a is required for the gene body methylation at Polycomb group (PcG) target developmental genes. DNMT3b has higher DNA methylation activity and hence a dominant role in the de novo methylation of X-chromosomes [19, 20]. Both DNMT3a and 3b function in conjunction with a third methyltransferase, DNMT3L. Although DNMT3L lacks methyltransferase activity, it acts as a cofactor regulating the activity of DNMT3a and b [21]. Maintenace methylase, DNMT1, maintains the methylation pattern set by the other two methyltransferases. DNMT1 maintains the methylation pattern through mitosis. After DNA replication, DNMT1 binds to the hemimethylated CpG sites and methylates the newly synthesized strand. Specific recruitment of DNMT1 to the hemimethylated sites is mediated through UHRF1, an E3 ubiquitin ligase [22]. Vertebrates have another DNMT, DNMT2, that shares high homology with other DNMTs. DNMT2 has a very poor/null methyltransferase activity on DNA templates. However, DNMT2 catalyzes tRNA methylation efficiently [23]. Methylation in the gene promoter represses transcription by 1) inhibiting the binding of different transcription factors (TFs) and/or RNA PolII to DNA, and 2) recruiting methyl binding proteins (MBPs), which bind to repressors and histone deacetylases [16, 24]. On the contrary, removal of the methyl group occurs passively during DNA replication, when the newly synthesized strand fails to add a methyl group [25]. Demethylation also occurs in an enzyme-mediated process. The methylated base is converted to a modified nucleotide by oxidation or deamination reaction catalyzed by ten-eleven translocations (TETs) and activation-induced deaminase (AID), respectively. The modified nucleotide is then recycled to generate cytosine by the base excision repair (BER) pathway [26].

## Histone modifications

Histone proteins form the framework upon which the DNA is bound. Two units each of H2A, H2B, H3, and H4 histones associate to form a core histone octamer. The octamer is bound by 147 bases of DNA to form a nucleosome, the fundamental unit of chromatin compaction. Nucleosomes remain connected in a “beads-on-a-string” pattern by linker histone (H1) and associated DNA, allowing easy access for the transcriptional machinery and higher gene activity. Such open regions in the chromatin are referred to as euchromatin. Heterochromatin refers to the organization of nucleosomes into tight bundles, reducing the access of the transcriptional machinery. Histone tails extending from the nucleosome surface, as well as the ones, present within the body of the octamer, serve as the sites for chemical modification. Additionally, histones present in the octamer core can be substituted by a variant. This opens up associated DNA causing their activation [27, 28]. Modification of histones through chemical processes can be done through post-translational addition or removal of methyl, acetyl, sumoyl, and phosphate. The modifications also include ubiquitination, ADP-ribosylation, deamination, and proline isomerization [29, 30]. The addition of any of these groups alters the charge associated with the histone molecule and hence its interaction with the negatively charged DNA. Thus, these modifications change the accessibility of TFs and cofactors to the associated DNA [31, 32]. The addition/removal of acetyl or methyl groups is the most common histone modification and is discussed below.

### Acetylation and deacetylation of histones

The addition of an acetyl group to the histone neutralizes the positive charge on the histones and reduces their attraction to the DNA molecules. This makes the DNA more accessible to the binding of TFs and other cofactor molecules, thereby positively affecting gene expression. Acetyl groups are added to the lysine residues present in the histone proteins. Acetylation is catalyzed by an enzyme called histone acetyltransferases (HATs/KATs) and is divided into two categories. Type *a* HATs are in the nucleus and they carry out the acetylation of nucleosomal histones and promote their transcription. Type *b* HATs are involved in the acetylation of newly synthesized histone molecules, before their incorporation into the nucleosome complex. They are distributed in the cytoplasm. Within the nucleus, histone acetylation can be reversed by HDACs. They remove the acetyl groups from the histone proteins, thus increasing the attraction of histone with the DNA molecule. This leads to the condensation of chromatin and hence gene repression. Eighteen HDACs identified in mammals have been classified into four different groups. Class I HDAC consists of the nuclear-localized HDAC1, 2, 3, and 8. HDACs shuttling between nucleus and cytoplasm constitute class II and include 4, 5, 6, 7, 9, and 10. Class III HDAC comprises NAD+ dependent proteins called sirtuins and class IV comprises HDAC11.

### Methylation and demethylation of histones

Histone methylation within the nucleus is controlled by histone methyltransferases and histone demethylases. Methyl groups from S-adenosyl methionine are transferred to the lysine or arginine residue present in H3 and H4 histone by histone methyltransferases. Depending on the residue getting methylated and the degree of methylation, their effect on gene expression can vary. The important sites of methyl group addition to a lysine on H3 are 4, 9, 27, and 36, and on H4 is 20. Generally, H3 methylation on the 4th (K4) or 36th (K36) lysine residue activates transcription, whereas K9 and K27 methylation repress genes. H3K4 me1 is often associated with enhancer regions [25, 33]. Another histone methyltransferase called disruptor of telomeric silencing-like (DOT1L) catalyzes H3K79 methylation [34]. Histone methylation is reversed by demethylases. Histone demethylase, Jumonji domain-containing protein 3 (Jmjd3) antagonizes the repression caused by H3K27me3 methylation during hypoxic conditions [35]. Jmjd6 is a histone arginine demethylase catalyzing H3R2 and H4R3 demethylation (Flt1; [36]). H3K4 di/trimethylation is reversed by jumonji AT-rich interactive domain 1B (JARID1B) and Lysine Demethylase 5B (KDM5B). However, H3K4me1 and H3K4me2 are removed by another demethylase, lysine-specific demethylase 1 (LSD1) [37]. Plant homeodomain finger protein 8 (PHF8) is a histone demethylase catalyzing the removal of methyl groups from histone 3 lysine 9 (H3K9) and H4K20 [38].

## Non-coding RNAs

Non-coding RNAs (ncRNAs) are a group of untranslated RNA molecules with regulatory functions. Based on the length of the RNA, ncRNAs are classified into small ncRNAs (sncRNAs) and long ncRNAs (lncRNAs). Small RNAs usually range in their size from 18 to 35 nucleotides, whereas the lnc RNAs are more than 200 nucleotides in length. SncRNAs show high functional variations and include transfer RNA (tRNA), ribosomal RNA (rRNA), small nuclear RNA (snRNA), small nucleolar RNA (snoRNA), Piwi-interacting RNA (piRNA). LncRNAs include intergenic ncRNAs, long intronic RNAs, telomeric ncRNA, pseudogene transcripts, enhancer RNA, and promoter-associated long RNA [39]. However, in the forthcoming sections, we will limit ourselves to ncRNAs involved in post-transcriptional regulation, directly by competing with functional RNAs. These include miRNAs and lncRNAs, specifically those binding to the target RNA and inhibiting translation. MiRNAs are single-stranded RNAs typically ranging in length from 20 to 24 nucleotides. They are located in the cytoplasm and stimulate the degradation of target RNA molecules by a pathway involving RNA induced silencing complex (RISC). The expression and function of miRNAs are closely associated with other epigenetic modifiers [39, 40]. LncRNAs show a greater variation in their sequence. They can also function as competitors to endogenous RNAs. LncRNA expressed from pseudogenes function as ‘antagomirs’ or ‘miR sponges’ by sequestering miRNAs [39, 41].

## Epigenetics of the blood–brain barrier

### BBB formation

During embryonic development, the mesoderm differentiates into angioblast and develops into a primitive blood vessel, this process is defined as vasculogenesis. Following this, new capillaries grow from this existing blood vessel by the process defined as angiogenesis. In mice, blood vessels invade the brain at embryonic day 9.5. It was reported that BBB genes including TJ proteins occludin and claudin-5 are expressed in the brain ECs at the initial stages of angiogenesis [42, 43]. However, a functional intact BBB is reported to be formed at E−15.5 in mice [44]. In *humans*, angiogenesis does not begin until fetal week 8, and the BBB is reported to be functional at an age of 4 months [45]. A detailed review of existing knowledge in the formation of BBB is available in [46, 47]. The details on vascular development in the brain and patterning are beyond the scope of this review, but it is relevant to the discussion in the review to provide a clear distinction between the neurovascular unit and BBB.

### Neurovascular unit

**Fig. 1:**
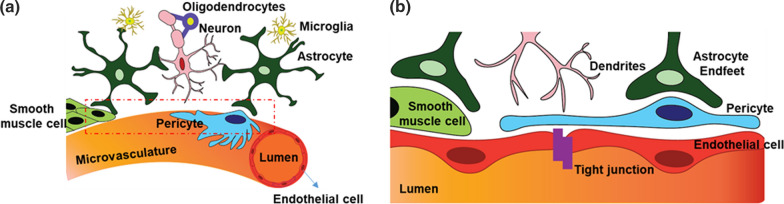
Diagrammatic representation of a neurovascular unit (NVU). NVU consisting of vascular cells, glial cells and neurons are shown in **a**, and the enlarged view of the inset is given in **b**. Endothelial cells (red), pericytes (blue), and smooth muscle cells (pale green) represent the vascular cells. The glia cells are given in dark green (astrocytes), and blue (oligodendrocytes), yellow (microglia) and the neurons in pink color. Tight junctions connecting the endothelial cells are also shown in **b**

As the blood vessel invades the brain, the ECs and perivascular cells, called pericytes, come to close contact with both neuronal and glial cells to form a neurovascular unit (NVU, Fig. 1) [48, 49]. The vascular components (ECs, pericytes, and vascular smooth muscle cells) and neuroglial components (neurons, oligodendrocytes, microglia, astrocytes, and astrocyte-derived basement membrane) of the NVU interact dynamically and communicate to regulate proper angiogenesis, BBB formation, and maintenance in the brain and the blood-retinal barrier (BRB) in the eye. This unique communication program likely provides important cues to modify the epigenetic programs in BBB cell types and thus support the formation and maintenance of BBB, until now these interactions have not been studied in any detail.

### Structural Components of the BBB

The specialized multicellular BBB structure contributes to the barrier properties including limiting or regulating the transport of molecules (influx and efflux), protection from toxic materials and pathogens (Fig. 1). CNS blood capillaries formed by ECs are the primary functional component of the BBB. Perivascular cells pericytes and ECs share a common basement membrane with attachment points to extracellular matrix components mediated by integrins and less ubiquitous cell-ECM receptors such as dystroglycan. The BBB is composed of a basement membrane (comprised of e.g. type IV collagen, laminin, and fibronectin), surrounded by astrocyte end-feet ensheathing the vessels and pericytes (PCs) [50]. In CNS vessels, ECs are held together with the help of strong tight junctions (TJs), which limit the paracellular flux of solutes, and are characterized by specific transporters, which,deliver molecules across the barrier and securely controls the brain homeostasis. The structural components of the BBB are comprehensively reviewed in [51–53]. Emerging experimental techniques in genomics and proteomics are now providing a larger picture of the molecular components of the BBB. Current understanding of the unique transcriptome and proteins in CNS ECs was obtained mainly by comparing isolated CNS ECs with peripheral and other brain cells [42, 43, 54–57].

## Tight junctions

Highly expressed TJ proteins are unique to CNS endothelial cells. It is the TJs between adjacent endothelial cells that confer the low paracellular permeability and high electrical resistance of the barrier, making it able to function 50–100 times tighter than peripheral microvessels [58–60]. TJs consist of complexes of claudins, occludins, and other transmembrane proteins scaffolded to the actin cytoskeleton by zona occludens (ZO) proteins [59, 61]. Together, these elements function to maintain homeostasis in the changing milieu of the CNS.

### DNA methylation

Currently, no data exist to explain the DNA methylation events that directly regulate the expression of TJ proteins. This study is only an example of indirect regulation of TJ localization/degradation via TIMP2 and MMPs, and no direct DNA-methylation regulation for TJ proteins has been reported until now. Following middle cerebral artery occlusion (MCAO) in mice, a global DNA methylation event was reported as an increase in global DNA methylation in the mouse brain. The study shows that these global methylation changes after MCAO increased the methylation in the promoter of tissue inhibitors of metalloproteinase 2 (TIMP2). TIMP2 functions to inhibit matrix metalloproteinase (MMPs) which are a group of enzymes involved in protein degradation of the extracellular matrix and the non-covalent binding of TIMPs will inhibit MMP activity. MMP-mediated disruption of TJ proteins is well documented [62, 63]. Increased MMP activity/secretion can affect BBB permeability, with MMP-2 and -9 being associated with BBB breakdown following stroke. Consequently, decreased TIMP2 expression/activity can be related to increased MMP-9 activity and degradation of the basal lamina [64] and tight junction components [65] resulting in BBB damage. Together, the MCAO-induced hypermethylation of the TIMP2 promoter contributes to a decreased activity of TIMP2 and an increased MMP activity that contributes to TJ protein degradation. Consistent with these findings, the pharmacological and genetic inhibition of DNMT was effective in attenuating the stroke symptoms [66, 67].

Conversely, a leaky BBB can contribute to changes in DNA methylation events leading to gene expression changes. Using in vitro and in vivo BBB disruption models, it was shown that BBB leakage can influence the expression of the DNA methyltransferase enzyme DNMT3b [68]. The study reported that BBB disruption influenced the DNA methylation events via increased expression of noncoding RNA miRNA29b. The increased miRNA29b affects the expression of DNMT3b and MMP expression. Supporting these findings, DNA methyltransferase inhibitor treatment (5-Azacytidine) ameliorates the BBB damage via reducing the expression of miRNA29b [68]

Future studies are required to investigate the role of DNA methylation changes in diseases where the BBB is compromised. Further studies are also warranted to elucidate how BBB leakage-induced stress contributes to DNA methylation events in other cell types of the neurovascular unit.

### HDACs and their inhibitors

Histone acetylation and deacetylation play a critical role in chromatin remodeling and epigenetics. HDACs have been identified as potential therapeutic targets in different neurological diseases [69]. The availability of different specific HDAC inhibitors has augmented our understanding of HDAC functions, mechanism of actions, and genomic profile influenced by this mechanism. Several compounds that inhibit HDAC activity have now been developed and characterized. Clinically HDAC inhibitors are successful in causing cell growth arrest, differentiation and/or apoptosis, and tumor growth restriction. Promisingly, the clinical efficacy of HDAC inhibitors extends beyond cancer treatments, and they have now been explored for their therapeutic potential in all top 10 leading causes of death in the US [70].

The most notable secondary effect after stroke is BBB damage. Recent efforts were made to target the HDACs to ameliorate TJ protein degradation. HDAC inhibitors, valproic acid, and sodium butyrate (class I, IIA, and III inhibitors) were used to treat an ischemic stroke rat model, and these effects on protecting the BBB were studied. It was reported that this treatment was beneficial in decreasing the degradation of TJ proteins such as Claudin-5 and ZO-1. The associated mechanism was through suppression of NF-*κ*B activation and MMP-9 induction [71, 72]. In another recent study, class IIA HDAC inhibitor—TMP269 treatment for mice subjected to cerebral ischemia/reperfusion injury leads to an increased expression of the tight-junction proteins, ZO-1, Occludin, and Claudin-5 in ECs, and thereby stabilize the BBB [73].

In parallel to this, recent research investigates the epigenetic changes after stroke. An increased HDAC expression was reported after an ischemic stroke. The author claims that this can contribute to BBB injury, and inhibiting HDAC can protect BBB [71, 74]. Conversely, an increased expression of HDAC4 after ischemic stroke was reported to protect the BBB break down via elevation of TJ proteins like claudin-5, occludin, and ZO-1, and through reducing the expression of NADPH oxidase and MMP-9 [75]. In an in vitro ischemic model using oxygen–glucose deprivation (OGD) in CNS endothelial cells an upregulation of endothelial HDAC9 expression was shown and this was associated with a decreased expression of TJ proteins, like ZO-1, claudin-5, and occludin. Supporting these findings, genetically targeting HDAC9 pre-OGD re-established the TJ protein expression in ECs [74]. Another in vitro study, using cultured primary human brain microvascular ECs undergoing OGD and reoxygenation, reported increased transendothelial cell permeability and downregulation of junctions proteins. These changes were correlated with increased HDAC3 activity and decreased PPARγ activity. Supporting the role of HDAC3 in regulating the TJ protein expression, treatment with a selective HDAC3 inhibitor RGFP966 reduced the paracellular permeability and increased the expression of TJ protein Claudin-5 via PPARγ receptor [76]. The same group also reported an increased HDAC3 expression in the hippocampus and cortex of diabetic mice accompanied by BBB leakage and was rescued via HDAC3 inhibitor treatment through miR-200a/Keap1/Nrf2 signaling pathway [77].

### Histone methylation

Another important and prevalent chromatin modification that is known to influence TJ protein expression is a post-translational modification of histones by methylation. A recent study showed that the glucocorticoid dexamethasone (glucocorticoids are currently used clinically for preventing tumor-associated brain edema [78]) can suppress the expression of JMJD3, a histone H3K27 demethylase in TNFα treated BBB disruption model on mouse brain microvascular endothelial cell line (bEnd.3). This happens via the recruitment of glucocorticoid receptor α (GRα) and nuclear receptor co-repressor (N-CoR) to the negative glucocorticoid response element in the upstream region of the JMJD3 gene. Further, the decreased expression of JMJD3 is correlated with a decreased activation of MMP-2, MMP-3, and MMP-9, and increased expression of claudin-5 and occludin [79–81]. Polycomb repressive complex proteins catalyze the methylation of H3K27 leading to repressive histone modification H3K27me3 in the gene promoter. A study to understand the role of vascular endothelial cadherins (VEC) in vascular stabilization, showed that VEC overexpression increases the expression of the claudin-5 gene by preventing the binding of PRC2 to the CLDN5 gene. VEC mediates this by forming a complex with Wnt transducer β-catenin and PRC2 subunit EZH2 [82]. *Caveolin-1* a key protein involved in the BBB [83–85] was repressed in Influenza-associated encephalopathy (IAE). The repression was mediated by the SET domain bifurcated 2 (Setdb2) by methylation of histone H3 lysine 9 [86].

From the above-discussed studies, it is evident that histone modifications have a critical role in controlling the expression of TJ proteins. However, more in-depth attempts are needed to understand the epigenetic mechanisms and signaling pathways that contribute to the formation and maintenance of TJs.

### Adherens junctions

The BBB is characterized by the high expression of TJs and low expression of adherens junctions (AJs) when compared to non-CNS EC barriers [87]. The basic molecular structure of adherens junctions (AJs) resemble TJs. A major AJ reported to be present in the CNS ECs is CDH5 along with a low expression of N- and E-cadherins [88]. It is reported that during CNS angiogenesis ECs have a relatively high expression of cadherin-10 compared to CDH5 [89, 90]. Stable AJs are key to the formation of TJs. It was reported that TJ protein *CLDN5* expression was upregulated by CDH5 by inducing the phosphorylation of forkhead box factor FoxO1 through Akt activation and by limiting the translocation of β-catenin to the nucleus [91]. Furthermore, the same group reported the epigenetic link for these findings (described in the TJs histone methylation section of this review).

## Transporters or solute carriers

The barrier to paracellular diffusion contributed by TJs potentially isolates the brain from many essential polar nutrients such as glucose and amino acids necessary for metabolism and therefore the CNS endothelium forming the BBB express a large number of specific transporters, including solute carriers and ABC (ATP-binding cassette) transporter proteins, for a wide variety of solutes and nutrients, mediating flux into and out of the brain [92–101]. Classification and roles of BBB transporters can be found in [102–104].

Glucose is the primary metabolic fuel for the mammalian brain and a continuous supply is required to maintain normal CNS function. The BBB regulates glucose transport into the brain via specific glucose transporters. A study to understand the prioritized glucose supply into the brain during fasting reports that fasting‐induced the production of ketone body β‐hydroxybutyrate (β‐OHB) which enhances expression of the glucose transporter gene *GLUT-1* via histone modifications. Brain microvascular endothelial cells treated with β‐OHB upregulated the expression of GLUT-1 via inhibiting HDAC2 and elevation of acetylation in H3K9 at the critical *cis*‐regulatory region [105]. The multidrug resistance protein 1 (MDR1, ABCB1, P-glycoprotein) is a major efflux transporter located on the surface of capillary endothelial cells that restricts the accumulation of xenobiotics in the brain. Immortalized human brain capillary endothelial (hCMEC/D3) cells treated with HDAC inhibitors valproic acid (VPA), apicidin, and suberoylanilide hydroxamic acid (SAHA) increased the mRNA and protein levels of MDR1 by 30–200% via increased acetylation in H3K9/K14 [106].

Apart from the above few studies, no other research was found reporting on the role of epigenetic programs in regulating the expression of BBB transporters.

## Other cell types in the BBB

### Pericytes

The abluminal surface of the CNS vessel shows the highest coverage of pericytes that invest and support the endothelial layer throughout the vasculature. Cerebral vessels have a high pericyte to EC ratio [107, 108] pointing to their significant role in the neurovascular unit, controlling BBB integrity and function, supporting the stability of vessels, contributing to the elasticity of vessels regulating the blood flow, and protecting endothelial cells from potentially harmful substances [109–111]. Crosstalk between pericyte and endothelial cells enhances the EC TJ formation and decreases transcytosis and leukocyte adhesion molecule expression in the developing BBB [43]. Considering the aforementioned functions, dysfunction or apoptosis of blood–brain barrier pericytes is a vital factor in the pathogenesis of several diseases that are associated with microvascular instability.

### HDACs and their inhibitors

An in vitro study was conducted to investigate the effect of HDAC inhibitors on pericyte proliferation, cell viability, migration, and differentiation. The results showed that HDAC inhibitors valproic acid and trichostatin A inhibited the proliferation and migration of pericytes with no effect on cell viability. Further, HDAC inhibitor treatment in pericytes increased the transcription of angiogenesis-related genes such as angiopoietin-like 4, transforming growth factor beta2, and TIMP2 [112]. BBB is also reported to be compromised during HIV infection. An attempt to investigate the role of occludin on BBB breakdown after HIV infection and its impact on pericytes reports that occludin levels control the metabolic responses of pericytes after HIV infection. HIV infection in pericytes reduces the occludin level and this is correlated with a decreased expression and activation of the class III histone deacetylase sirtuin (SIRT)-1 along with elevated nuclear localization of gene repressor C-terminal-binding protein (CtBP)-1 and NFκB-p65 activation. Further, this study demonstrates occludin as a NADH oxidase and showed that cellular levels of NADH inversely correlated with the cellular content of occludin that controls the expression and activation of SIRT-1 [113]**.** In another study, overexpression of SIRT3 was shown to increase the pericyte density and improved the pericyte EC coverage in the lungs of LPS treated mice [114]. The overexpression of SIRT3 could also be promising for brain pericytes and to support BBB repair after an injury.

## Astrocytes

Astrocytes serve as a bridge that connects neuronal signaling to the CNS vasculature. Astrocyte structure includes specialized processes called astrocyte endfeet that extend from the astrocyte cell body and attach to the basement membrane that surrounds the endothelial cells and pericytes [115–117]. Astrocytes regulate the BBB through its synaptic glutamate levels, via scavenging free radicals and producing neurotrophic factors to communicate with other cell types in the BBB [118, 119]. In an in-vitro co-culture experiment with ECs cultured alone, ECs co-cultured with astrocytes or astrocyte-conditioned media enhanced the endothelial cell barrier properties including the transporter expression and increased TJ formation thus supporting the astrocyte interaction with endothelial cells supporting the formation of the BBB [120].

### DNA methylation

Astrocyte end-feet have orthogonal arrays of intramembranous particles (OAPs) consisting of the most abundant water channel aquaporin-4 (AQP4) and the ATP-sensitive inward rectifier potassium channel Kir4.1 [121]. It is reported that DNA methylation is an important process in the development of astrocytes since demethylation of astrocyte-specific genes such as GFAP, S100β, and AQP4 in neural stem cells (NSCs) promotes the switch from neurogenesis to astrogenesis [122–125]. In astrocytes, changes in global DNA methylation patterns have been shown to occur in psychiatric disorders [126] and alcohol abuse [127]. However, DNA methylation events in astrocytes that contribute to BBB formation or damage are not known.

### HDACs and their inhibitors

The GLUT1 transporter has an important role in astrocyte metabolism and supporting neuronal energy metabolism [128]. It is reported that cerebral astrocyte culture incubated with Pan-HDAC inhibitor valproic acid could increase histone acetylation at the SLC2A1 promotor thereby facilitating glucose uptake in the astrocytes [129]. This is important as maintaining the astrocyte metabolism after an injury can ensure BBB maintenance. Addressing the expression pattern of HDACs in the cortex and hippocampus of mice after photothrombotic infarction showed that HDAC1 was expressed in the nuclei and cytoplasm of GFAP(+) astrocytes in the hippocampus. Expression was also observed, to some extent, in astrocyte end-feet [130]. In another study, the activity of HDAC2 and HDAC8 in neurons and astrocytes was reported to be elevated 7 days after ischemia. The study also reports that HDAC2 was predominantly localized in the nuclei, and HDAC8 was predominantly observed in the cytoplasm. Together the above research can be read as the increased expression of HDACs have some critical role in BBB damage and HDAC inhibitor can potentially minimize this effect after ischemic stroke [71, 74, 131].

## BBB and non-coding RNA

MicroRNAs (miR) which cause degradation or translational repression of mRNA play an important role in the development and progression of BBB dysfunction. Knowledge of miRNAs function in the BBB came from the early work identifying miR-125a-5p and other miRNAs in regulating brain endothelial tightness [132]. The increasing number of research articles connecting different miRNAs and BBB disruption highlights the significance of targeting miRs for BBB repair in various neurological conditions including TBI and stroke [133, 134]. Keeping in mind that small nucleotide-based drugs are easy to develop and targeting miR has great success, drugs targeting and based on miRs have a pronounced potential in treating BBB damage in neurological diseases [133].

Table 1 shows different miRs and their mechanism of action which are reported to be involved in the BBB disruption after different pathological conditions including stroke. Our impression from the literature is that miRs and BBB disruption are studied mostly in stroke conditions. Although miRNAs are inhibitory, depending on their target mRNA, an increase or decrease of miRNA expression is relevant. MiRs can directly or indirectly cause the degradation of BBB proteins or translational repression of BBB mRNA. For example, it is reported that miR-132 can directly target MMP-9 and after stroke, the reduced miR-132 expression increases MMP-9 activity which degrades TJ proteins or components of the basal lamina. Further TJ proteins like ZO-1, occludin, and claudin-5 were reported to be positively regulated by miR-126, miR-107, and miR-127 and negatively regulated by miR-98 and miR-150 in different disease models. A comprehensive, systematic review of miRNA regulation of TJ proteins has been conducted [135], and more miRNA have been found involved in TJ protein regulation summarized in Table 1.

**Table 1:** MicroRNA candidates which are reported being involved in BBB disruption

miRNA	Disease model	Species	miRNA Expression	BBB dysfunction	Mechanism
miR-107 [160]	Alzheimer's disease	In vitro human brain ECs(HBMEC)	Decreased	amyloid-β incubation down-regulates the expression of tight junction proteins ZO-1, Occludin, and Claudin-5	Overexpression protects BBB Via its direct target endophilin-1
miR-126-3P [161]	Intracerebral hemorrhage	Rat	Decreased	Cerebral edema, BBB leakage	Administration of miR-126 protects BBB via targeting PIK3R2 and the Akt signaling pathway
miR-126-3P, 5P [162]	Stroke	Mice	Decreased	Brain edema, BBB leakage, decreased ZO-1 and Occludin	miR-126-3p, 5p overexpression reduced the expression of proinflammatory cytokines IL-1β and TNF-α and adhesion molecules VCAM-1 and E-selectin and attenuated BBB disruption
miR-1303 [163]	Coxsackievirus A16 (CA16) infection model	In vitro and Monkey	Decrease	Degradation of junctional complexes Claudin4, Claudin5, CDH5, and ZO-1	Infection downregulates miR-1303, which directly targets MMP9
miR-130a [164]	Stroke	Rat	Increase	Brain edema, BBB leakage	Increased miR-130a expression after stroke directly inhibit Homeobox A5 expression, which down-regulates occludin
miR-132 [165]	Stroke	Mice	Decreased	Brain edema, BBB leakage	miR-132 overexpression decreased the degradation of tight junction proteins CDH5 and β-Catenin via targeting MMP9
miR-132 [166]	Stroke	Mice	Increased	Brain edema, BBB leakage, decreased expression of CDH5 and β-catenin	Targets MMP-9 and suppress the expression
miR-143 [167]	methamphetamine-induced BBB dysfunction model	Mice/in vitro HBMEC	Increased	The decreased expression of TJ proteins including Claudin-5, ZO-1, and Occludin	miR-143 targets p53 upregulated modulator of apoptosis (PUMA) to induce BBB dysfunction
miR-149-5p [168]	Stroke	Mice	Decreased	BBB leakage, Pericyte migration	Targeting sphingosine-1-phosphate receptor (S1PR)2 expressed in pericytes
miR-150 [169]	Stroke	Rat	Increased	BBB leakage and decreased claudin-5 expression	miR-150 could regulate the claudin-5 expression and endothelial cell survival by targeting Tie-2
miR-155 [170]	Experimental autoimmune encephalomyelitis (EAE)	Mice/ in vitro HBMEC	Decrease	BBB leakage	Negative regulator of BBB targeting cell adhesion components annexin-2 and claudin-1 and focal adhesion components DOCK-1 and syntenin-1
miR-21 [171]	Stroke	Rat	Increase	Brain edema, BBB leakage	Target MAPK signaling
miR-210 [172]	neonatal hypoxic-ischemic encephalopathy	Rat	Increased	cerebral edema, BBB leakage, reduced expression of tight junction protein occludin and adherens junction protein β-catenin	Increased level of miR-210 in the model negatively regulates BBB integrity
MiR-212/132 [173]	Hypoxia	Mice/ in vitro HBMEC	increased	Increased expression of MiR-212/132 in hypoxic HBMEC decreased mRNA and protein expression of Cldn1, Jam3, and Tjap1	By targeting Cldn1, Jam3, and Tjap1
miR-29b [174]	Stroke	Mice	Decrease	BBB leakage, Decreased expression of ZO-1, and occludin	Via targeting AQP4
miR29b [68]	BBB dysfunction model	Mice/in vitro	increased	Homocysteine induced BBB leakage	miR29b-mediate BBB dysfunction via targeting DNMT3b and MMP9
miR-501-3p [175]	Vascular dementia	Mice	Increase	Decreased expression of claudin-5, ZO-1, and occludin	TNFα upregulates the miR-501-3p that directly targets ZO-1
miR-539 [176]	Stroke	Rat	Decrease	BBB leakage	miR-539 targets MMP-9
miR-98 [177]	Stroke	Mice	Decrease	BBB leakage,	Reduced leukocytes infiltration and diminished microglia activation
miRNA‐9‐5p [178]	TBI	Rat/In vitro	Increased	Decreased expression of claudin-5, ZO-1, and occludin	Via activating Hedgehog pathway and inhibiting NF‐κB/MMP‐9 pathway

In an effort to investigate the differences in miRNAs expression in rat cortical pericyte during hypoxic stress showed differential regulation of miRNAs with 27 miRNAs upregulated and 31 miRNAs downregulated. These differentially regulated miRNAs are capable of targeting important signaling factors such as HIF-1α (miR-322 [136] increased and miR-199a [137] decreased in pericytes after hypoxic stress), TGF-β (miR-376b-3p [138] increased miR-140[139], miR-145 [140] decreased in pericytes after hypoxic stress) and VEGF (miR-126a [141] increased, and miR-297[142], miR-16[143], miR-17-5p[144] decreased in pericytes after hypoxic stress). Let-7 miRNA expression in pericytes is reported as involved in pericyte differentiation in response to hypoxic stress [145]. As an example of endothelial-pericyte cross talk, an *in-vitro* study reports that pericytes could uptake miR-503 originated from endothelial cells exposed to high glucose (hyperglycemia) [146, 147]. Under diabetic-induced microvascular dysfunction, inhibition of lncRNA-myocardial infarction-associated transcript (MIAT) or lncRNA-metastasis-associated lung adenocarcinoma transcript 1 (MALAT1) is shown to reduce the pericyte loss [148].

Astrocytes are known to express several miRNAs and these miRNAs can affect various functions of astrocytes [149–154]. Astrocytes-derived factors such as vascular endothelial growth factors, matrix metalloproteinases, nitric oxide, and endothelin-1 can affect the vascular tone and BBB permeability [155–159]. Considering this astrocytic miRNAs are a potential therapeutic target for BBB damage however this axis is yet to be explored.

## Epigenetic strategies for the treatment of blood–brain barrier damage

Currently, there are no treatment modalities available to directly treat BBB dysfunction. The most common drugs to treat BBB dysfunction are glucocorticoids which help to stabilize TJ proteins [179–182]. In multiple sclerosis, interferon-beta treatment is one of the most promising immunomodulatory for reducing inflammatory damage. Few pre-clinical and clinical evidence support that interferon-beta could also be effective in treating BBB [183–185]. However, these drugs are not specific to the BBB, are mainly used as anti-inflammation, affect numerous physiologic processes, and increase the risk of complications, including infection and hyperglycemia [186]. The gene expression profile of humans and mice that contributes to the BBB function is now available [187–192]. This dataset was acquired by comparing the brain EC transcriptome to peripheral ECs [187, 193]. A deep understanding of the epigenetic mechanisms that regulate the transcription of BBB genes in brain ECs and other BBB cell types is warranted so that epigenetic drugs can be repurposed or developed to manipulate the BBB gene expression to treat BBB dysfunction in various neurological diseases. Epigenetic markings of DNA and histones are introduced and removed by enzymes, and they are therefore potentially reversible, paving the way for potential therapeutic targets. Promisingly small compounds that target epigenetic mechanisms including HDAC inhibitors have been FDA approved for the treatment of certain cancers [194]. Although many epigenetic drugs are in clinical trials for cancer treatments (Table 2), there is still room for improvement, as they are relatively unstable, can have toxic side effects, and are not available for oral administration. Table 2 mentions the available compounds that target epigenetic mechanisms. Information on epigenetic drugs' clinical availability and mechanism of action can be found in [194–198]. Promisingly, apart from its use as cancer drugs, clinically HDAC inhibitors are now used in psychiatry and neurology as mood stabilizers and anti-epileptics [199].

**Table 2:** Details of the drug targeting epigenetic modifiers in endothelial cells

Name of the drug	Epigenetic enzyme altered by the drug	Status of the clinical study/disease or condition
Trichostatin A	Inhibits class I and II HDAC enzymes	Clinical trial-completed (NCT03838926)/Relapsed or Refractory Hematologic Malignancies
Valproic acid/VPA	Inhibits class I and class IIa HDACs	Clinical trial-completed (NCT01233609)/ Retinitis Pigmentosa
Sodium butyrate/NaB	Inhibits class I and class IIa HDACs	Clinical trial-completed NCT00800930/ Shigellosis
Suberoylanilide hydroxamic acid/SAHA/Vorinostat	Inhibits class I and II HDACs	Clinical trial-completed (NCT00106626)/ Advanced Cancer
3-Deazaneplanocin-A /DZNep	Inhibits EZH2 methyltransferase	–
Methylthioadenosine/MTA	Inhibits H3K4 methylase	Clinical trial-completed (NCT03083015)/ Necrotic Pulp
Morpholino	Inhibits histone acetyltransferase 7/KAT7	Clinical trial-completed (NCT03375255)/ Muscular Dystrophy, Duchenne
Resveratrol/RV	Activates sirtuin1/Sirt1	Clinical trial-completed (NCT01010009)/ Cognitive and Cerebral Blood Flow Effects of Resveratrol
Panobinostat/LBH589	Inhibits ClssI, II, and IV HDACs	Clinical trial-completed (NCT00840346)/ Acute Myeloblastic Leukaemia
Entinostat/SNDX-275/MS 27–275	Inhibits HDAC1/3	Clinical trial- completed (NCT02897778)/Cardiac Safety Study With Advanced Solid Tumors
Mocetinostat	Inhibits HDAC1 and HDAC2	Clinical trial-completed (NCT02303262)/ Metastatic Leiomyosarcoma
Lithium	Inhibits GSK3b	Clinical trial-completed (NCT01259388)/ Progressive Multiple Sclerosis
5-Aza-2′-deoxycytidine (5-dAzaC)	Inhibits DNMT	Clinical trial-completed (NCT00744757)/ Myelodysplastic Syndrome
GSK2879552	Inhibits LSD1	Terminated clinical trials Relapsed/Refractory Small Cell Lung Carcinoma
Tazemetostat	Inhibits EZH2 (PRC2 subunit)	Clinical trial-completed (NCT02860286)/ Relapsed or Refractory Malignant Mesothelioma

Emerging questions related to the prognostic and diagnostic value of epigenetic modifications in the BBB genes for predicting neurodegenerative processes and cognitive decline now exist. To learn the gene expression programs in CNS ECs that contribute to BBB dysfunction, the gene expression profile of brain ECs from different mice disease models which shows BBB dysfunction such as stroke, TBI, multiple sclerosis were compared. Interestingly, though the trigger for BBB dysfunction in each disease differs, a similar change in EC gene expression pattern contributes to BBB dysfunction [188]. This is a very significant finding as we can correlate the gene expression changes to changes in the epigenetic marks on those BBB genes. Furthermore, by manipulating the epigenetic regulation of those genes, it is possible to manipulate the expression of BBB genes to repair the BBB damage. Numerous studies have shown that AD brain endothelium expresses low levels of GLUT1, a BBB-specific glucose transporter, which then leads to reduced transport of glucose into the brain [200]. Identifying these epigenetic changes and reversing them may offer a promising therapeutic opportunity to target BBB dysfunction in AD.

For human BBB studies currently, MRI imaging is the most commonly used technique. Limited availability of human brain vessels for BBB studies makes it almost impossible to understand the BBB rupture mechanism in humans. Advances in stem cell technology now allow developing in vitro human BBB models from patients with different neurodegenerative disorders carrying genetic mutations [201, 202]. These human studies will enhance the knowledge of epigenetic variations in several neurological diseases that leads to BBB damage by comparing it to in vitro BBB models from healthy or isogenic controls.

## Conclusion and future direction

BBB leakage is a major factor that determines disease progression, outcome, and therapeutic response. Management and prevention of BBB leakage pose a notable challenge to the medical community. Currently, no readily available clinical agent exists that can effectively prevent leakage or repair BBB. Novel therapeutic strategies that can meet this challenge might emerge from understanding the epigenetics of BBB development and damage. From our above review of literature, it is clear that most mechanistic insights on epigenetics and BBB breakdown have been gained from animal models of stroke. We have limited knowledge about the epigenetic mechanisms underlying breakdown in neurodegenerative disorders such as Alzheimer’s disease and multiple sclerosis. More research is warranted to investigate the epigenetic changes in BBB genes of CNS ECs, pericytes, astrocytes, and neurons after a BBB breakdown. We hope the development of advanced sequencing techniques, availability of ChiP antibodies and advanced methods to purify the brain cell types will allow comprehensive research in this area. Furthermore, there is a huge lack of human data to support the preclinical findings. Considering species differences affecting BBB permeability, it is also very relevant to investigate that the existing animal data are translatable to the human situation.

## Data Availability

Data sharing not applicable to this article as no datasets were generated or analyzed during the current study.
